# A Novel Sit4 Phosphatase Complex Is Involved in the Response to Ceramide Stress in Yeast

**DOI:** 10.1155/2013/129645

**Published:** 2013-09-04

**Authors:** Alexandra Woodacre, Museer A. Lone, Daniel Jablonowski, Roger Schneiter, Flaviano Giorgini, Raffael Schaffrath

**Affiliations:** ^1^Department of Genetics, University of Leicester, Leicester, LE1 7RH, UK; ^2^Division of Biochemistry, Department of Biology, University of Fribourg, CH-1700 Fribourg, Switzerland; ^3^Institut für Biologie, FG Mikrobiologie, Universität Kassel, 34132 Kassel, Germany

## Abstract

Ceramide is a building block for complex sphingolipids in the plasma membrane, but it also plays a significant role in secondary signalling pathways regulating cell proliferation and apoptosis in response to stress. Ceramide activated protein phosphatase activity has been previously observed in association with the Sit4 protein phosphatase. Here we find that *sit4*Δ mutants have decreased ceramide levels and display resistance to exogenous ceramides and phytosphingosine. Mutants lacking *SIT4* or *KTI12* display a shift towards nonhydroxylated forms of long chain bases and sphingolipids, suggesting regulation of hydroxylase (*SUR2*) or ceramide synthase by Sit4p. We have identified novel subunits of the Sit4 complex and have also shown that known Sit4 regulatory subunits—SAP proteins—are not involved in the ceramide response. This is the first observation of separation of function between Sit4 and SAP proteins. We also find that the Sit4p target Elongator is not involved in the ceramide response but that cells deficient in Kti12p—an accessory protein with an undefined regulatory role—have similar ceramide phenotypes to *sit4*Δ mutants. Therefore, Kti12p may play a similar secondary role in the ceramide response. This evidence points to a novel Sit4-dependent regulatory mechanism in response to ceramide stress.

## 1. Introduction

Ceramide is a building block for complex sphingolipids which comprise an important structural component of the plasma membrane. It is also a secondary signalling molecule that accumulates in response to stresses such as heat shock [1]. It is therefore important for sphingolipid metabolism to be tightly regulated, and the damaging effects of dysregulation are apparent in patients with Tay-Sachs disease, Fabry disease, and other inherited sphingolipidosis disorders [2]. Ceramide mediates controlled cell death by triggering several signalling cascades to initiate caspase-dependent and independent apoptosis [3]. In contrast, the phosphorylated ceramide precursors dihydrosphingosine (DHSP) and phytosphingosine (PHSP) are signals for pathways that promote cell proliferation [4].

Although it is known that the cellular response to ceramide is important for the regulation of cell proliferation and cell death pathways, the precise molecular mechanisms for this regulation still remain elusive. It is vital to further understand the way cells respond to stress in order to develop strategies to modify them, either to accelerate cell death using targeted anticancer drugs or to prevent accumulation of toxic products in sphingolipidoses [5–7].

**Figure 1:**
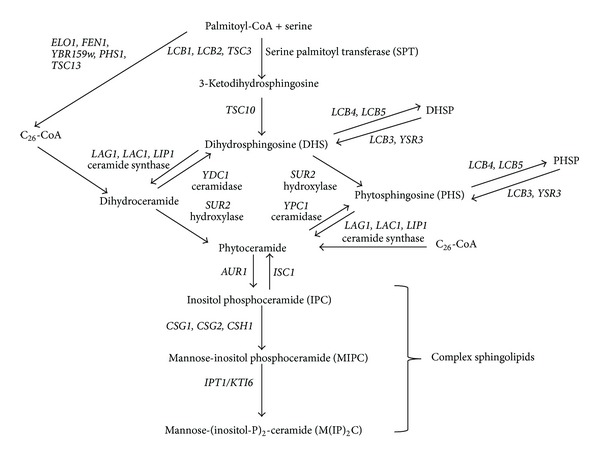
Biosynthesis of sphingolipids in *Saccharomyces cerevisiae.* Key enzymes discussed in the text are highlighted and genes encoding all relevant parts of the pathway are included. The directions of arrows indicate the end products of enzymatic reactions.

*Saccharomyces cerevisiae* has been used effectively as a model to study sphingolipid metabolism, and Figure 1 shows a detailed summary of the sphingolipid biosynthetic pathway in yeast [8]. Many of the genes involved are conserved from yeast to higher eukaryotes, with diversion in the synthesis of complex sphingolipids occurring only after the production of ceramides, resulting in the production of different end products in the pathway. The addition of inositol to ceramide in yeast forms inositol phosphoceramide, and glucose, galactose or phosphorylcholine is added to ceramide in mammalian cells to generate glycosphingolipids and sphingomyelin [9].

Early work by Nickels and Broach showed that a ceramide-activated phosphatase activity was present in *Saccharomyces cerevisiae*, which is separate from the activity of the major PP2A phosphatases Pph21p and Pph22p [10]. The ceramide resistance of a *sit4*Δ mutant strain suggested that the PP2A-like phosphatase Sit4p is responsible for this activity. *SIT4* is an essential gene in the absence of the suppressor allele *SSD1-v* and has important roles in the progression of the cell cycle, cell integrity, nutrient responses via TORC1, drug resistance via efflux pumps, and tRNA modification [11–16]. Diverse regulatory subunits of Sit4p are partially responsible for the different specificities of Sit4p; for example, Tap42p is phosphorylated by Tor and binds Sit4p [17] and Sap185p and Sap190p subunits are essential for correct phosphoregulation of Elongator and tRNA modification [18]. Mutation of the SAPs (Sit4 associated proteins) can confer different specificities on Sit4p but a deletion of all four SAPs always results in the same phenotypes as deletion of *SIT4*, for example resistance to the tRNAse toxin zymocin, cell cycle arrest, and sensitivity to rapamycin [19, 20]. The accessory protein Kti12p is also essential for the phosphoregulation of the Elongator subunit Elp1p by the Sit4p/Sap185p/Sap190p complex. Kti12p interacts with the casein kinase Hrr25p in an Elongator-dependent manner but the mechanism by which Kti12p regulates phosphorylation remains unclear [21].

The aim of this study was to further investigate the role of Sit4 as the ceramide-activated protein phosphatase (CAPP) in yeast. We identify *KTI12* as an important gene mediating ceramide toxicity and show that ceramide toxicity is independent of Elongator function. Mutants lacking *SIT4* or *KTI12* have decreased levels of ceramide and the balance of hydroxylated and nonhydroxylated sphingolipids is altered. The confirmation that Tpd3p and Cdc55p can interact with Sit4p and a separation of function between *SIT4* and the regulatory SAP subunits indicates that the CAPP is likely to be an alternative Sit4 complex operating via a novel mechanism.

## 2. Methods

### 2.1. Yeast Strains and Media

Yeast were routinely grown in yeast extract peptone dextrose medium (YPD; 1% yeast extract, 1% peptone, 2% glucose) at 30°C with shaking. Glucose was replaced with 2% galactose to induce expression of *SIT4* and *PPH21* from the *GAL1* promoter. Synthetic defined medium without inositol (0.67% yeast nitrogen base, 2% glucose, supplemented with essential amino acids) was used for labelling with [^3^H]*myo*-inositol. Yeast strains used in this study are listed in Table 1.

### 2.2. Growth Tests Using Ceramide and Long Chain Bases

C2-ceramide, C2-phytoceramide, dihydrosphingosine (DHS), and phytosphingosine (PHS) powders were purchased from Enzo Life Sciences and resuspended in 100% ethanol. Stock solutions (5 mg/mL) were stored at −20°C for a maximum of 1 week. Yeast cultures were diluted from a starter culture to 5 × 10^3^ cells/mL in YPD containing ceramides/long chain bases or an equal volume of ethanol as an untreated control. Cultures were grown until the untreated control reached exponential phase (from 15–36 hours depending on the strain) and the OD_600_ measured for both treated and untreated cultures. After 24 hours, an additional dose of ceramide/long chain base was added to counteract the effects of compound degradation. The amount of growth in each concentration of ceramide/long chain base was then expressed as a percentage of the growth in untreated media. This method of standardising growth enables the comparison of slow-growing mutants such as *sit4*Δ to a faster-growing wild-type strain. Raw OD_600_ data is provided in Supplementary Tables 1 and 2 (see Supplementary Material available online at http://dx.doi.org/10.1155/2013/129645). A minimum of three biological replicates were performed for each strain and a one-way ANOVA with Bonferroni post-test was used to determine if growth was significantly different from the wild type (CY4029).

### 2.3. Immunoprecipitation

Dynabeads (Invitrogen) were coupled with 5 *μ*g of anti-HA antibody per mg of beads, following the manufacturer's instructions. Total protein extracts were prepared from 50 mL cultures grown for 8 hours in YPD supplemented with galactose. Cell pellets were resuspended in 400 *μ*L B60 buffer (50 mM HEPES pH 7.3, 60 mM sodium acetate, 5 mM magnesium acetate, 0.1% Triton X-100, 10% glycerol, 1 mM sodium fluoride, 20 mM glycerophosphate, 1 mM DTT, 1X Complete Mini Protease Inhibitor Cocktail (Roche)). An equal volume of glass beads was added and cells disrupted using a bead beater for 1 minute, followed by centrifugation at 15700 g, 4°C for 5 minutes. The supernatant was transferred to a new tube and centrifuged at 15700 g, 4°C for 20 minutes. The cleared protein extract was quantified using spectrophotometry and 3.5 mg of total protein extract was incubated with 1.5 mg of antibody-coated beads for 30 minutes at 4°C. Unbound proteins were removed by three washes with 1 mL B60 buffer, and antibody-bound proteins were eluted with 50 *μ*L of 10% (v/v) SDS for 10 minutes at room temperature. The beads were then removed with a magnet and the supernatant used for Western blot analysis. SDS-PAGE of 100 *μ*g of total protein from each strain and immunoprecipitation supernatants (equal volumes) was carried out using 12% acrylamide gels and then Western blotted at 100 V for 1 hour. Blots were probed with anti-HA (F7 Santa Cruz), anti-c-myc (A14 Santa Cruz), or anti-Tpd3 (Y. Jiang, University of Pittsburg School of Medicine, USA) and secondary antibodies conjugated to horseradish peroxidase (Roche Diagnostics) were detected by chemiluminescence and exposed to X-ray film.

### 2.4. Sphingolipid Analysis by ESI-MS

Overnight cultures grown at 24°C in YPD media were diluted to OD_600_ 0.2 and grown until they reached OD_600_ of 2. A total of 10 OD units of cells were collected and washed once with sterile water. Lipid extraction was performed by a two-step lipid extraction method [23]. Cells were resuspended in 1 mL of 150 mM ammonium bicarbonate (NH_4_HCO_3_) and 600 *μ*L of glass beads were added. After cell lysis using a Precellys 24 homogenizer ((Bertin technologies) 5000 rpm, 3x 30 sec on 30 sec off), lysates were diluted in 5 mL of 150 mM NH_4_HCO_3_ solution and internal standards were added. Long chain bases and ceramides were quantified relative to respective lipid standards, and inositol phosphoceramides were measured relative to a phosphoinositol standard. Lipid standards were purchased from Avanti Polar Lipids. ESI-MS analysis was performed using a Bruker Esquire HCT ion trap mass spectrometer in positive or negative ion mode. Peaks were identified based on their fragmentation pattern and by comparison to commercially available standards. Three biological replicates were included in each analysis.

### 2.5. Incorporation of [^3^H]-Labelled Inositol

Overnight cultures grown at 24°C in YPD were diluted to OD_600_ 1.0 in synthetic defined media containing 40 *μ*Ci [^3^H]  *myo*-inositol (American Radiolabelled Chemicals, MO, USA) and grown at 24°C for 4 h until they reached OD_600_ of approximately 2. A total of 10 OD units were harvested, and lipids were extracted using chloroform/methanol/water (10 : 10 : 3) and analysed as previously described [24] by thin layer chromatography with or without mild-base treatment. Mild-base treatment to remove inositol phosphate and leave only N-acetylated sphingolipids was performed by incubating lipids in 0.1 M NaOH at 30°C for 1 hour. Radioactivity was detected using a phosphorimager (Typhoon FLA9500, GE Healthcare) and a representative image of two biological replicates is shown.

## 3. Results

### 3.1. Deletion of Sit4-Associated Proteins (SAPs) Does Not Confer Resistance to Exogenous Dihydroceramide

**Figure 2:**
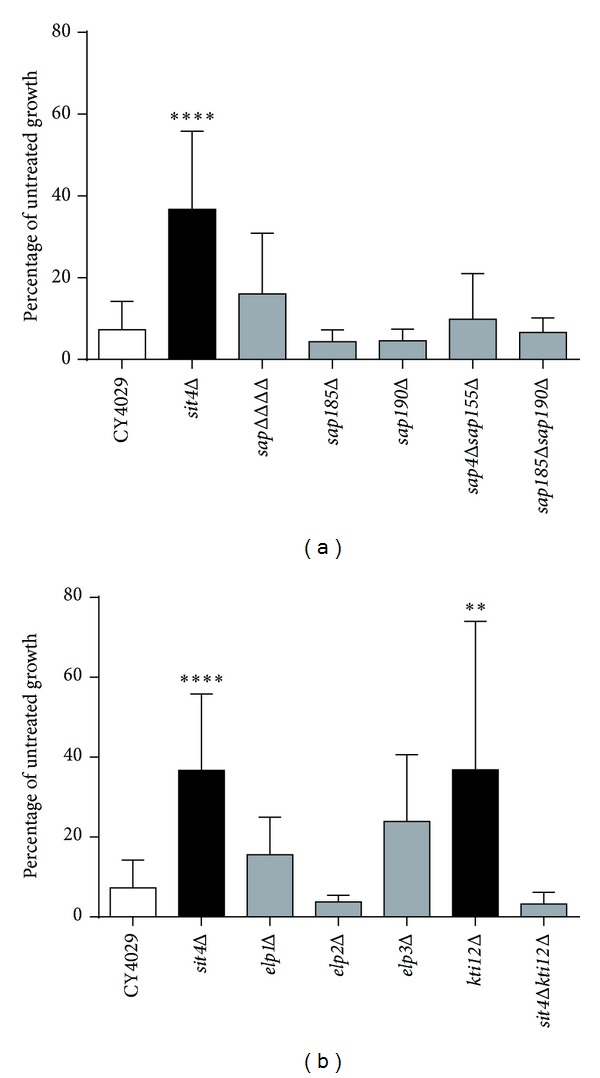
Deletion of *SIT4* or *KTI12* confers resistance to excess dihydroceramide. (a) Ceramide growth tests in Sit4 associated protein (SAP) mutants. (b) Ceramide growth tests in Elongator-associated mutants. Yeast cultures were diluted to 5 × 10^3^ cells/mL in YPD with the addition of either 15 *μ*M C-2 dihydroceramide or an equal volume of ethanol. Cells were grown until the untreated culture reached exponential phase and the OD_600_ of all cultures was determined. Growth in 15 *μ*M dihydroceramide is expressed as a percentage of untreated growth. Raw OD_600_ values are given in Supplementary Table 1. A minimum of three replicates are shown and error bars represent the standard deviation above and below the mean. A one-way ANOVA with a Bonferroni post-test was used to determine if mutants showed a significant difference in growth compared to the wild type (CY4029) (**P < 0.01, ***P < 0.001, and ****P < 0.0001).

As previously described [10], deletion of *SIT4* leads to significant resistance to 15 *μ*M dihydroceramide (Figure 2, *P* < 0.0001). However, mutation of the four SAP regulatory proteins, either individually or in combination, does not confer resistance to dihydroceramide (Figure 2(a)). In previous studies, the phenotype of the quadruple *sap* mutant has been indistinguishable from that of *sit4*Δ [19, 20]. Thus, the ceramide sensitivity of the *sap* mutant is the first observed separation of function between *sit4*Δ and *sap*ΔΔΔΔ.

### 3.2. Kti12p Appears to Be the Only Elongator-Associated Protein Involved in the Ceramide Response

As Sit4p plays a major role in the phosphoregulation of the Elongator complex [18, 21] and previous studies suggested that Elongator mutants were resistant to ceramide, Elongator components were investigated as potential targets of Sit4p in the response to excess dihydroceramide. Although deletion of Elongator subunits did not confer statistically significant resistance to 15 *μ*M dihydroceramide, deletion of the Elongator accessory protein Kti12p did confer resistance (Figure 2(b)) to some extent, though the obtained data were rather variable (Supplementary Table 1). Interestingly, deletion of *SIT4* and *KTI12* in tandem restored sensitivity to dihydroceramide, whereas in a previous study mutants lacking one or both of these genes had the same phenotype that resulted in hyperphosphorylation of Elp1p and zymocin resistance [18]. In addition, phosphorylation of Elp1p was unchanged in the presence of dihydroceramide, and this was not affected by deletion of *SIT4* and/or *KTI12* (data not shown). Therefore, our data suggest that Kti12p might play a regulatory role in the ceramide response that is independent of Elongator.

### 3.3. PHS Resistance of *sit4* **Δ **Indicates Separation of Function from *kti12*Δ

**Figure 3:**
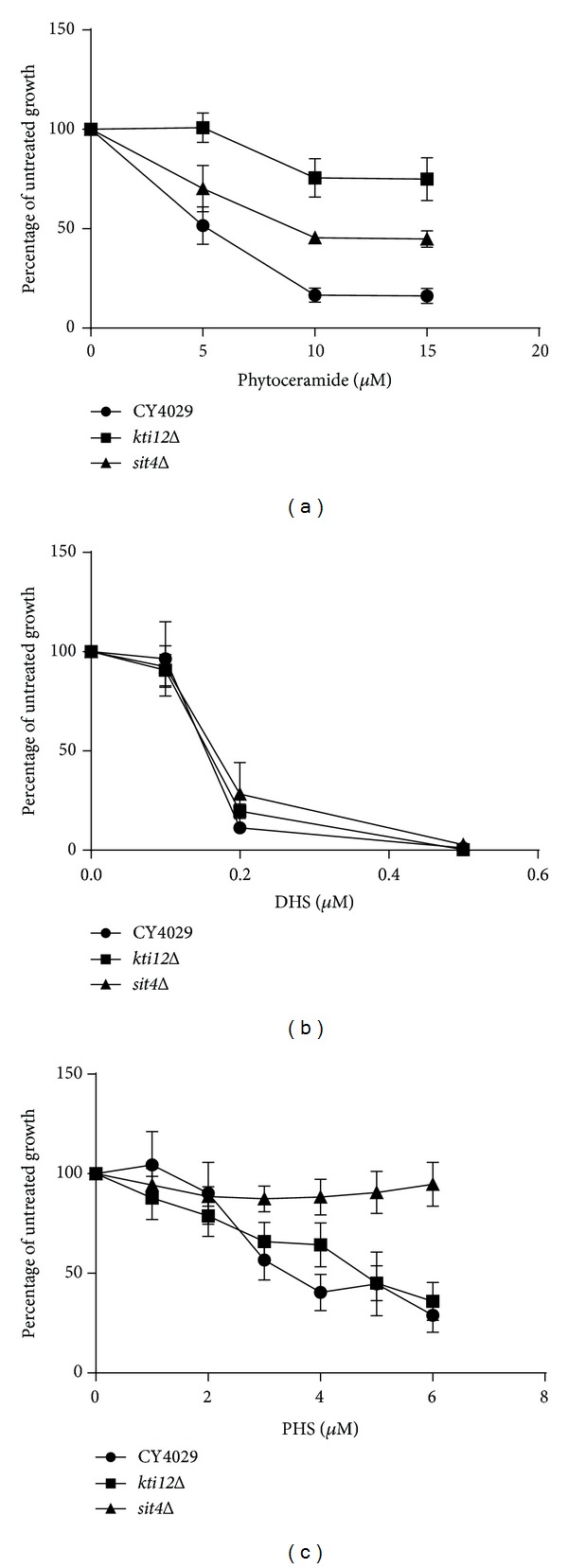
Response of *sit4*Δ and *kti12*Δ mutants to phytoceramide and long chain bases. Yeast cultures were diluted to 5 × 10^3^ cells/mL in YPD with the addition of the indicated concentrations of (a) phytoceramide, (b) dihydrosphingosine (DHS), (c) phytosphingosine (PHS), or an equal volume of ethanol. Cells were grown until the untreated culture reached exponential phase and then the OD_600_ of both treated and untreated cells was measured and plotted as a percentage of untreated growth. Raw OD_600_ values are given in Supplementary Table 2. A minimum of three replicates are shown and error bars represent the standard error above and below the mean. A Student's *t*-test was used to determine if the mutants showed a significant difference in growth compared to the wild type (CY4029) at each concentration shown.

Growth in phytoceramide decreases in a concentration-dependent manner in both wild-type and mutant strains; however, *sit4*Δ and *kti12*Δ mutants show significantly more growth (*P* < 0.005) than the parental CY4029 strain at concentrations of 10–15 *μ*M (Figure 3(a)). In contrast, growth of *sit4*Δ and *kti12*Δ mutants in excess dihydrosphingosine (DHS) is indistinguishable from CY4029 (Figure 3(b)). The most striking result is that while *kti12*Δ is also sensitive to phytosphingosine (PHS), *sit4*Δ shows significant (*P* < 0.05) resistance to 3–6 *μ*M PHS (Figure 3(c)), suggesting divergence of function between Kti12p and Sit4p in the response to long chain bases.

### 3.4. Ceramide and Long Chain Base Levels Are Reduced in *sit4*Δ and *kti12*Δ Mutants

**Figure 4:**
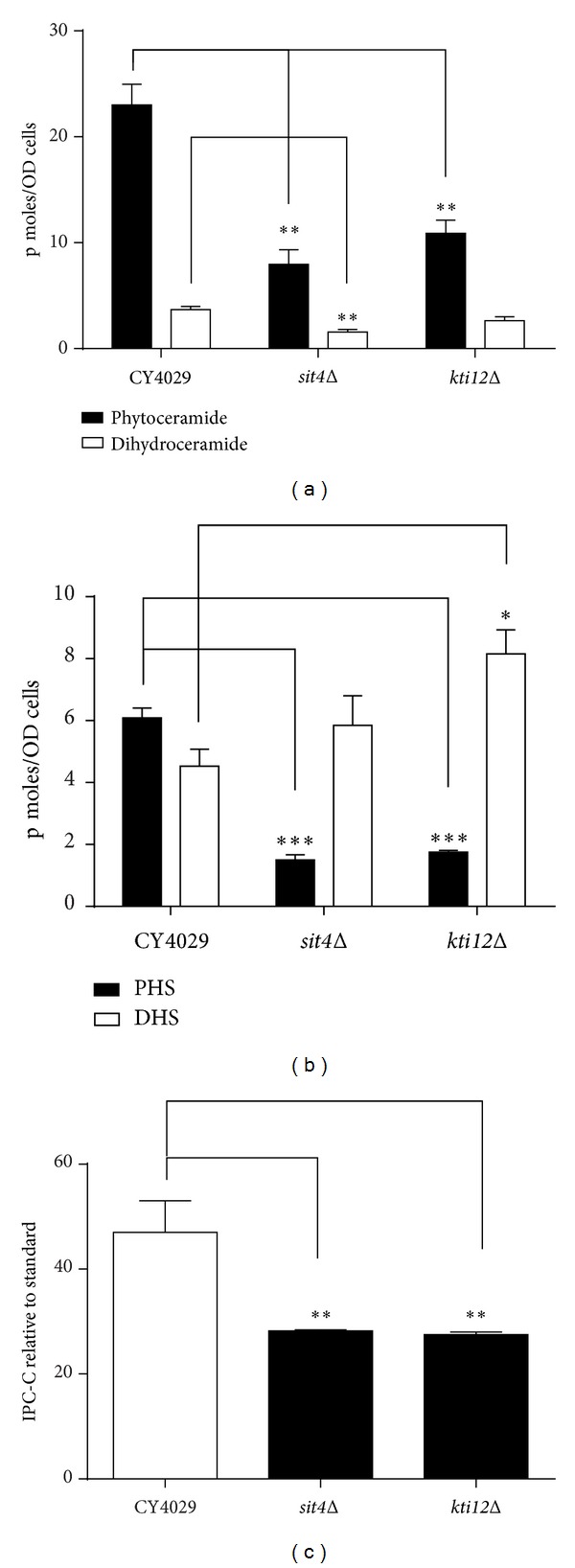
Mass spectrometric analysis of sphingolipid species. Yeast cultures were diluted to an OD_600_ of 0.2 in YPD and grown for 8 hours at 24°C. A total of 10 OD_600_ units of cells were removed and lipids extracted for mass spectrometry analysis. (a) Ceramides, (b) long chain bases phytosphingosine (PHS) and dihydrosphingosine (DHS), and (c) Inositol phosphoceramide-C (IPC-C) were quantified using relevant internal standards. Average values for a minimum of three biological replicates are shown and error bars represent the standard error above and below the mean. A Student's *t*-test was used to determine if the mutants showed a significant difference from the wild type CY4029. (*P < 0.05, **P < 0.01, and ***P < 0.005).

To investigate the possibility that Sit4p and Kti12p regulate the sphingolipid biosynthesis pathway, we measured steady state levels of ceramides, long chain bases, and inositol phosphate in *sit4*Δ and *kti12*Δ strains relative to wild-type yeast cells. Deletion of *SIT4* or *KTI12* reduces the intracellular levels of phytoceramide by approximately 50% (Figure 4(a)). Levels of dihydroceramide are also reduced in both mutants, but the decrease is only statistically significant in *sit4*Δ (Figure 4(a)). This suggests that the mutants may be able to tolerate otherwise toxic levels of exogenous ceramides due to the constitutively lower levels present within the cell. The reduction of PHS levels by approximately two-thirds in the *sit4*Δ mutant could permit the strain to survive excess concentrations of PHS (Figure 4(b)). However, a similar decrease in PHS levels in the *kti12*Δ mutant does not correlate with resistance to exogenous PHS (Figures 4(b) and 3(c)), suggesting that a more complex mechanism underlies PHS resistance. There is a small increase in the levels of DHS in *sit4*Δ and *kti12*Δ mutants (Figure 4(b)) which is unlikely to affect the toxicity of DHS seen in Figure 3(b).

### 3.5. *Sit4* Mutants Show an Increase in the Proportion of Dihydro Sphingolipids and a Corresponding Decrease in the Proportion of Hydroxylated Sphingolipids

**Figure 5:**
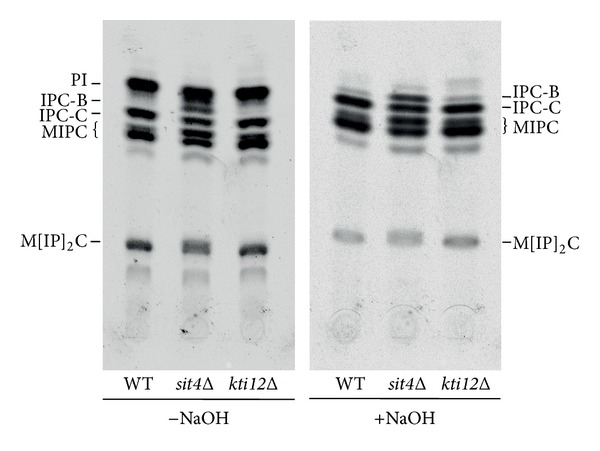
Tritium labelled inositol incorporation into yeast cells. CY4029 (WT), *sit4*Δ, and *kti12*Δ were incubated with [^3^H]-inositol for 4 hours, and lipids were extracted and analysed by thin layer chromatography, before and after mild-base treatment to remove inositol phosphate (PI). Equal CPMs were loaded for all the samples. A representative image of two biological replicates is shown.

Tritium labelled inositol incorporation was used to analyse the maturation of complex sphingolipid species formed from both dihydroceramide and phytoceramide. Dihydroceramide B′ (18:0;2/26:0;0) and phytoceramide C (18:0;3/26:0;0) form inositol phosphoceramide B (IPC-B) and inositol phosphoceramide C (IPC-C) respectively. IPC-C and the corresponding MIPC-C generated from it form the relatively more abundant species of their sphingolipid class in the wild type (Figure 5). Interestingly, the *sit4*Δ mutant contains increased levels of IPC-B and MIPC-B compared to the wild type, with a decrease in the levels of IPC-C and MIPC-C (Figure 5). The *kti12*Δ mutant also shows a similar trend in the relative levels of sphingolipid species, but the differences from the wild type are less pronounced than those for *sit4*Δ. This indicates a shift towards more sphingolipids being synthesised from dihydroceramide/DHS precursors than from the hydroxylated phytoceramide/PHS precursors.  Figure 4(c) provides additional evidence for this shift and quantification of IPC-C levels shows a significant decrease in both *sit4*Δ and *kti12*Δ mutants. This also correlates with the increase in DHS and decrease in PHS levels observed in *sit4*Δ and *kti12*Δ mutants (Figure 4(b)).

### 3.6. Novel Interactions of Sit4p with Tpd3p and Cdc55p Suggest a Role for an Alternative Phosphatase Complex in the Response to Ceramide

**Figure 6:**
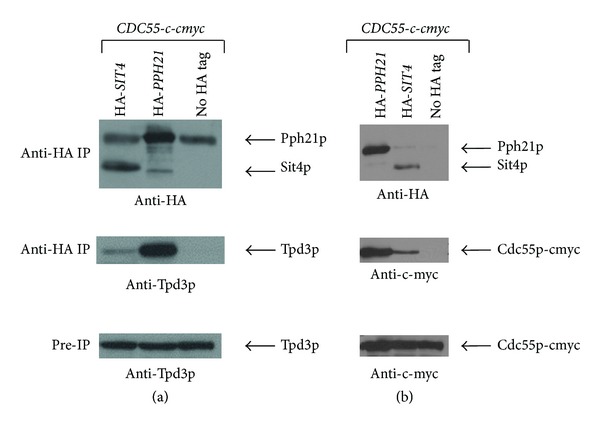
Immunoprecipitation of HA-Sit4p reveals novel interactors. Equal amounts of protein extracts were immunoprecipitated with magnetic beads coated with anti-HA antibodies, and the precipitates were then subjected to Western blotting and probed with anti-HA and anti-Tpd3 (a) or anti-c-myc (b) antibodies. Equal amounts of total protein extracts (without immunoprecipitation) were also probed with anti-Tpd3 or anti-c-myc antibodies.

Previous studies suggested that Tpd3p and Cdc55p could be part of the CAPP complex as deletion of these genes conferred resistance to ceramide [10]. Indeed, we found via immunoprecipitation experiments with HA-labelled Sit4p that Tpd3p and Cdc55p-c-myc interact with Sit4p, forming a minor complex compared to the Pph21p/Tpd3p/Cdc55p complex (Figure 6). As this novel Sit4p/Tpd3p/Cdc55p trimer is formed constitutively and is not induced by the presence of ceramide (data not shown), the mechanism by which the phosphatase is activated in response to an increase in ceramide levels remains unclear.

## 4. Discussion

The aim of this study was to further investigate the role of the Sit4 phosphatase in response to ceramide and to determine if this signalling pathway is directly related to the biosynthesis of ceramides and sphingolipids. The phosphorylation status of Orm1p regulates the activity of serine palmitoyltransferase and therefore the production of all downstream products of the sphingolipid pathway. Orm1p is phosphorylated by Ypk1p and evidence suggests that dephosphorylation may involve Sit4p and/or its TOR-dependent subunit Tap42p [25, 26]. However, Orm1p is unlikely to be the direct substrate for the Sit4p or Tap42p phosphatases as mutation of these genes leads to decreased phosphorylation of Orm1p [25].

The Sit4 phosphatase is a well-characterised regulator of tRNA modification via the Elongator complex [18, 21, 27–29]. However, here we show that the role of Sit4p in the ceramide response is independent of Elongator yet still involves the multifunctional and Elongator-related accessory protein Kti12p. Although previous work suggested that Elongator may be involved in ceramide resistance [18], more detailed analysis in this current study indicates that Elongator mutants are sensitive to ceramide. Although Kti12p is essential for the phosphoregulation of Elongator, its precise role remains unclear [18, 21]. Kti12p also has diverse roles in other cellular processes including the cell cycle [30] and transcription [31], and regulation of the ceramide response can now be added to this list. 

The Sit4 phosphatase has multiple regulatory subunits including the Sit4 associated proteins (SAPs) Sap4p, Sap155p, Sap185p, and Sap190p. A quadruple deletion of all SAPs is sensitive to excess ceramide, in contrast to the resistant *sit4*Δ mutant. Importantly, this is the first separation of function observed between the *sit4*Δ and *sap*ΔΔΔΔ mutants. This suggests that an alternative Sit4 phosphatase complex is involved in the regulation of the ceramide response, supporting the idea that this process is independent of Elongator functions that require Sit4/Sap complexes. The identification of a Sit4p/Tpd3p/Cdc55p trimer also supports the theory that the ceramide activated protein phosphatase could be acting via a previously unknown mechanism. 

The alteration of the sphingolipid makeup in *sit4*Δ and *kti12*Δ mutants and the decreased levels of ceramide and long chain bases indicate that there is regulation of the biosynthetic pathway at some level by Sit4p and/or Kti12p. The decreased level of endogenous ceramide and PHS in the mutants presumably enables them to survive an otherwise toxic concentration of these compounds. This suggests that deletion of *SIT4* and *KTI12* mediates a downregulation or partial inactivation of ceramide synthesis rather than a complete block, as there are clearly sufficient precursors available for effective biosynthesis of sphingolipids. The presence of multiple genes encoding enzymes for synthesis and degradation of ceramides is a key way in which sphingolipid metabolism can be maintained when the pathway is partially blocked. Phosphoregulation of ceramide synthases has not been previously observed, but three phosphorylated serine residues are conserved in both Lag1p and Lac1p ceramide synthases and could be potential targets for dephosphorylation by Sit4p [32, 33]. In common with *sit4*Δ and *kti12*Δ mutants, *lac1*Δ*lag1*Δ mutants are resistant to the tRNase toxin zymocin, but the mechanism of action is due to a defect in plasma membrane integrity caused by decreased levels of the sphingolipid M(IP)_2_C and not via Elongator [34]. 

In *sit4*Δ and *kti12*Δ mutants, the relative proportion of lipids synthesised from dihydroceramides/DHS is higher than those synthesised from phytoceramides/PHS, suggesting that there could be a defect in the Sur2 hydroxylase which hydroxylates both long chain bases and ceramides [35]. This is also reflected in the increased levels of DHS seen in the mutants and is therefore unlikely to simply be a defect in the synthesis of ceramide or downregulation at an earlier stage in the pathway, as not all components of the pathway are downregulated. The ceramidases Ypc1p and Ydc1p also have a minor ceramide synthase activity and show specificity for hydroxylated and nonhydroxylated forms of long chain bases, respectively. [36] Dysregulation in *sit4*Δ could cause a shift towards the synthesis of nonhydroxylated sphingolipids by these enzymes. However, these enzymes contribute a minor level of ceramide synthase activity compared to Lag1p, Lac1p, and Lip1p [37], so a change in their regulation is unlikely to have any detrimental effects on the overall sphingolipid composition of the plasma membrane, even if the balance of individual components is altered.

These new insights into the novel ceramide-associated functions of Sit4p and Kti12p are helpful in understanding the diverse roles these proteins play in the cell and expand our knowledge of their importance beyond their association with the Elongator complex. Relatively little is known about the human orthologues of Sit4p and Kti12p, and thus yeast studies are vital in unravelling the essential role they play in regulating cell proliferation and cell death in both healthy and malignant cells. 

## 5. Conclusions

This study indicates that the roles of Sit4p and Kti12p in the ceramide response are distinct from their roles in the regulation of the Elongator complex and are therefore likely to be mediated via a novel mechanism. The separation of function between *sit4*Δ and *sap*ΔΔΔΔ mutants and the interaction of Sit4p with the alternative regulatory subunits Cdc55p and Tpd3p also support this theory. Alterations in the levels of ceramides, long chain bases, and complex sphingolipids in *sit4*Δ and *kti12*Δ mutants indicate that these proteins are also likely to regulate the sphingolipid biosynthesis pathway. Future work will be targeted at delineating the underlying mechanism(s) underlying these observations.

## Supplementary Material

Supplementary Tables 1 and 2 include OD_600_readings and % growth values for all data points in Figures 2 and 3 respectively.

## Figures and Tables

**Table 1:** Yeast strains used in this study.

Strain	Genotype	Reference
CY4029	Mat a *ade2-1 his3-11,15 leu2-3,112 trp1-1 ura3-1 can1-100 SSD1-v1 gal+ *	[20]
CY3938	CY4029, *sit4*Δ*::HIS3 *	[20]
CY5236	CY4029, *sap4*Δ*::LEU2 sap155*Δ*::HIS3 sap185*Δ*::ADE2 sap190*Δ*::TRP1 *	[20]
CY5220	CY4029, *sap4*Δ::*LEU2 sap155*Δ::*HIS3 *	[20]
CY5224	CY4029, *sap185*Δ::*ADE2 sap190*Δ::*TRP1 *	[20]
CY4917	CY4029, *sap185*Δ*::ADE2 *	[20]
CY4380	CY4029, *sap190*Δ*::TRP1 *	[20]
DJY101	CY4029, *sit4*Δ*::HIS3 kti12*Δ*KlLEU2 *	[18]
LFY3	Mat a *ade2-1 his3-11,15 leu2-3,112, ura3-1 can1-100, elp1*Δ*::TRP1 *	[18]
LFY4	Mat a *ade2-1 his3-11,15 leu2-3,112, ura3-1 can1-100, elp2*Δ*::TRP1 *	[18]
LFY5	Mat a *ade2-1 his3-11,15 leu2-3,112, ura3-1 can1-100, elp3*Δ*::TRP1 *	[22]
LFY6	Mat a *ade2-1 his3-11,15 leu2-3,112, ura3-1 can1-100, kti12*Δ::*TRP1 *	[22]
AWY1	CY4029 *TRP1::GAL1::(HA)*_3_ *-SIT4 CDC55-*(*c-myc*)_3_:*:HIS3MX6 *	This study
AWY2	CY4029, *kanMX6::PGAL1::(HA)*_3_ *-PPH21, CDC55-(c-myc)*_3_ *::HIS3MX6 *	This study
AWY3	CY4029, *CDC55-(c-myc)*_3_ *::HIS3MX6 *	This study
