# Supplementary material for: A Novel Sit4 Phosphatase Complex Is Involved in the Response to Ceramide Stress in Yeast
Source: Oxid Med Cell Longev. 2013 Sep 4;2013:129645. doi: 10.1155/2013/129645 (PMC3777123; doi:10.1155/2013/129645)
Supplement: Supplementary file 1 — Supplementary Tables 1 and 2 include OD600readings and % growth values for all data points in Figures 2 and 3 respectively. [file 129645.f1.pdf]

Supplementary Table 1

Figure 2

CY4029	**0 µM Ceramide OD_{600}**	**15 µM Ceramide OD_{600}**	**% of untreated growth**
	0.63	0.03	4.11
	0.85	0.05	6.10
	0.87	0.06	6.82
	1.07	0.01	0.94
	1.08	0.02	1.94
	1.08	0.04	3.70
	1.21	0.00	0.00
	1.27	0.04	3.39
	1.48	0.02	1.22
	1.62	0.36	22.28
	1.96	0.09	4.69
	2.07	0.24	11.50
	2.68	0.23	8.62
	2.80	0.12	4.18
	2.94	0.12	4.08
	3.07	0.04	1.40
	3.91	0.52	13.30
	-	-	6.10
	-	-	7.95
	-	-	16.07
	-	-	25.52

sit4 Δ	**0 µM Ceramide OD_{600}**	**15 µM Ceramide OD_{600}**	**% of untreated growth**
	0.30	0.21	68.09
	0.35	0.06	18.34
	0.41	0.21	50.24
	0.49	0.14	28.98
	0.53	0.29	54.37
	0.55	0.22	40.55
	0.55	0.31	55.46
	0.65	0.08	12.31
	0.65	0.34	52.84
	0.69	0.26	37.83
	0.71	0.34	47.69
	0.82	0.13	15.73
	0.87	0.07	8.51
	1.16	0.11	9.40
	1.88	0.44	23.51
	1.89	1.39	73.55
	3.89	1.58	40.62
	-	-	24.04
	-	-	26.92
	-	-	29.02
	-	-	52.84

sap ΔΔΔΔ	**0 µM Ceramide OD_{600}**	**15 µM Ceramide OD_{600}**	**% of untreated growth**
	0.26	0.04	14.51
	0.65	0.32	49.23
	0.79	0.07	8.71
	0.92	0.07	7.61
	0.97	0.12	12.41
	1.13	0.09	7.52
	-	-	12.41

sap185 Δ	**0 µM Ceramide OD_{600}**	**15 µM Ceramide OD_{600}**	**% of untreated growth**
	1.20	0.04	2.92
	1.80	0.02	1.11
	3.42	0.22	6.43
	4.15	0.30	7.13

sap190 Δ	**0 µM Ceramide OD_{600}**	**15 µM Ceramide OD_{600}**	**% of untreated growth**
	0.91	0.03	2.75
	0.95	0.03	3.05
	1.81	0.14	7.90

sap4 Δsap155 Δ	**0 µM Ceramide OD_{600}**	**15 µM Ceramide OD_{600}**	**% of untreated growth**
	0.67	0.03	3.88
	0.74	0.05	6.76
	0.93	0.33	35.16
	1.54	0.08	5.46
	1.63	0.11	6.50
	2.21	0.15	6.61
	3.02	0.13	4.24

sap185 Δsap190 Δ	**0 µM Ceramide OD_{600}**	**15 µM Ceramide OD_{600}**	**% of untreated growth**
	0.47	0.03	6.81
	0.60	0.06	10.33
	0.68	0.03	5.00
	0.69	0.09	12.32
	0.96	0.02	2.47
	2.56	0.10	3.71
	3.02	0.17	5.63

elp1 Δ	**0 µM Ceramide OD_{600}**	**15 µM Ceramide OD_{600}**	**% of untreated growth**
	0.64	0.11	16.56
	0.80	0.23	28.50
	2.48	0.22	8.71
	-	-	6.42
	-	-	8.25
	-	-	24.91

elp2 Δ

0 µM Ceramide OD_{600}	15 µM Ceramide OD_{600}	% of untreated growth
0.40	0.02	5.82
0.48	0.03	5.21
0.67	0.01	2.09
-	-	1.53
-	-	3.75
-	-	4.10

elp3 Δ

0 µM Ceramide OD_{600}	15 µM Ceramide OD_{600}	% of untreated growth
0.69	0.31	44.20
1.69	0.10	5.86
2.80	0.21	7.46
-	-	20.50
-	-	21.97
-	-	43.33

kti12 Δ

0 µM Ceramide OD_{600}	15 µM Ceramide OD_{600}	% of untreated growth
0.82	0.89	108.54
1.22	0.19	15.25
1.59	0.66	41.38
-	-	10.94
-	-	12.31
-	-	32.41

sit4 Δ*kti12* Δ

0 µM Ceramide OD_{600}	15 µM Ceramide OD_{600}	% of untreated growth
0.40	0.03	7.50
0.97	0.01	1.03
1.19	0.03	2.52
2.00	0.04	2.00

Supplementary Table 2

Figure 3

OD_{600}

Phytoceramide (µM)

				**CY4029**			
**0**	0.76	0.84	1.51	1.59	1.99	2.76	
**5**	0.24	0.60	0.33	0.65	1.48	1.93	
**10**	0.02	0.11	0.24	0.37	0.41	0.69	
**15**	0.00	0.12	0.23	0.33	0.41	0.74	
				***kti12* Δ**			
**0**	0.57	0.63	1.20	1.71	2.52	2.59	2.79
**5**	0.57	0.52	1.52	2.19	2.49	2.15	2.40
**10**	0.31	0.44	0.74	0.69	2.46	2.89	2.60
**15**	0.28	0.37	0.70	0.77	2.99	2.61	2.72
				***sit4* Δ**			
**0**	0.33	0.50	0.54	0.57	0.97	1.02	1.03
**5**	0.16	0.50	0.24	0.20	1.05	0.58	1.02
**10**	0.12	0.23	0.30	0.21	0.49	0.46	0.51
**15**	0.10	0.26	0.25	0.25	0.62	0.36	0.46

% of untreated growth

Phytoceramide (µM)

				**CY4029**			
**0**	100.00	100.00	100.00	100.00	100.00	100.00	
**5**	30.84	71.39	21.92	40.94	74.37	69.93	
**10**	1.97	12.63	15.76	23.52	20.75	24.86	
**15**	0.00	14.42	15.10	20.75	20.35	26.63	
				***kti12* Δ**			
**0**	100.00	100.00	100.00	100.00	100.00	100.00	100.00
**5**	100.00	83.02	126.67	128.07	98.81	83.01	86.02
**10**	54.91	69.84	61.67	40.47	97.62	111.58	93.19
**15**	49.65	58.41	58.08	45.26	115.08	100.77	97.49
				***sit4* Δ**			
**0**	100.00	100.00	100.00	100.00	100.00	100.00	100.00
**5**	47.27	99.40	44.90	35.31	108.15	56.86	99.12
**10**	34.85	46.60	54.73	36.36	50.67	44.71	50.15
**15**	29.39	51.00	46.01	43.18	63.88	35.10	44.88

OD_{600}

DHS (µM)			**CY4029**			
**0**	0.76	1.32	1.44	1.53	1.74	4.62
**0.1**	1.32	1.32	1.30	1.22	1.79	1.47
**0.2**	0.10	0.31	0.08	0.16	0.22	0.15
**0.5**	0.01	0.00	0.00	0.01	0.05	0.10
			***kti12* Δ**			
**0**	1.35	1.99	2.01	2.47	3.00	3.09
**0.1**	1.32	1.77	1.98	2.96	2.13	2.10
**0.2**	0.38	0.45	0.25	0.73	0.55	0.22
**0.5**	0.02	0.02	0.00	0.00	0.00	0.00
			***sit4* Δ**			
**0**	0.27	0.39	0.57	0.70	0.77	1.00
**0.1**	0.25	0.32	0.35	0.66	1.07	0.83
**0.2**	0.03	0.01	0.00	0.00	0.59	0.80
**0.5**	0.02	0.01	0.00	0.00	0.00	0.05

% of untreated growth

DHS (µM)			**CY4029**			
**0**	100.00	100.00	100.00	100.00	100.00	100.00
**0.1**	173.68	100.00	90.28	79.74	102.87	31.82
**0.2**	13.42	23.41	5.21	10.33	12.41	3.16
**0.5**	1.45	0.00	0.00	0.65	2.76	2.21
			***kti12* Δ**			
**0**	100.00	100.00	100.00	100.00	100.00	100.00
**0.1**	97.78	88.94	98.51	119.84	71.00	67.96
**0.2**	28.00	22.61	12.54	29.55	18.23	7.25
**0.5**	1.19	0.80	0.00	0.00	0.00	0.00
			***sit4* Δ**			
**0**	100.00	100.00	100.00	100.00	100.00	100.00
**0.1**	95.09	82.44	61.95	93.46	138.96	83.00
**0.2**	11.32	1.78	0.00	0.00	76.36	80.30
**0.5**	9.06	3.05	0.00	0.00	0.00	5.00

PHS (µM)	OD_{600}						
				**CY4029**			
**0**	1.02	1.09	1.24	1.77	1.82	1.88	2.24
**1**	0.65	1.80	2.00	1.53	1.03	1.50	1.98
**2**	0.65	0.68	1.90	2.39	0.76	1.78	1.85
**3**	0.13	0.68	0.92	1.71	0.73	0.92	1.41
**4**	0.08	0.49	0.80	0.84	0.14	0.86	1.46
**5**	0.04	0.35	0.70	0.73	0.05	0.91	2.87
**6**	0.02	0.32	0.84	0.65	0.03	0.72	0.60
				***kti12* Δ**			
**0**	0.68	0.82	0.85	0.92	0.94	2.37	2.73
**1**	0.36	0.75	0.66	0.95	0.50	2.63	3.48
**2**	0.28	0.91	0.73	0.79	0.41	2.00	2.77
**3**	0.20	0.54	0.52	0.70	0.36	2.22	2.63
**4**	0.26	0.52	0.44	1.03	0.25	1.72	2.35
**5**	0.14	0.33	0.41	0.31	0.22	1.91	1.93
**6**	0.14	0.10	0.37	0.10	0.25	1.70	1.82
				***sit4* Δ**			
**0**	0.41	0.42	0.47	0.69	0.70	0.72	0.77
**1**	0.56	0.42	0.44	0.68	0.80	0.65	0.72
**2**	0.43	0.41	0.38	0.67	0.74	0.61	0.71
**3**	0.44	0.38	0.34	0.63	0.84	0.55	0.68
**4**	0.40	0.40	0.37	0.69	0.74	0.58	0.96
**5**	0.51	0.40	0.58	0.65	0.79	0.58	0.68
**6**	0.62	0.39	0.42	0.67	0.79	0.67	0.65
**0**	0.79	0.92	0.92				
**1**	0.25	0.83	0.85				
**2**	0.42	0.85	0.82				
**3**	0.35	0.87	0.85				
**4**	0.13	0.82	0.88				
**5**	0.05	0.88	0.82				
**6**	0.09	1.02	0.84				

% of untreated growth

**PHS (µM)**				**CY4029**			
**0**	100.00	100.00	100.00	100.00	100.00	100.00	100.00
**1**	63.92	165.14	161.29	86.44	56.76	79.79	88.39
**2**	63.53	62.29	153.23	135.03	40.11	94.68	82.59
**3**	12.25	62.11	73.79	96.61	40.12	49.15	62.95
**4**	7.75	44.77	64.52	47.46	7.69	45.48	65.18
**5**	3.53	31.65	56.69	41.07	2.86	48.56	128.13
**6**	1.96	29.72	67.58	36.89	1.81	38.24	26.79
				kti12 **Δ**			
**0**	100.00	100.00	100.00	100.00	100.00	100.00	100.00
**1**	52.50	90.63	77.29	103.05	53.09	110.97	127.47
**2**	40.44	110.71	85.76	86.04	43.19	84.39	101.47
**3**	29.41	66.55	60.82	76.66	37.77	93.67	96.34
**4**	37.79	62.90	51.65	112.43	26.81	72.57	86.08
**5**	20.00	40.15	47.76	33.59	22.87	80.59	70.70
**6**	19.85	12.29	43.65	10.69	26.60	71.73	66.67
				sit4 **Δ**			
**0**	100.00	100.00	100.00	100.00	100.00	100.00	100.00
**1**	137.16	100.95	94.02	97.68	114.90	90.93	93.39
**2**	105.87	97.14	80.77	96.24	106.59	85.50	91.57
**3**	106.36	91.65	71.58	90.59	119.63	76.01	87.81
**4**	98.04	94.75	78.21	99.57	105.87	80.75	124.90
**5**	123.47	94.75	123.72	93.63	113.04	80.20	87.81
**6**	151.10	93.56	90.38	96.96	112.75	93.44	83.92
**0**	100.00	100.00	100.00				
**1**	31.81	89.50	92.42				
**2**	52.85	80.95	88.20				
**3**	44.74	94.05	91.45				
**4**	16.86	88.31	95.24				
**5**	5.83	95.13	88.32				
**6**	11.91	110.71	101.95				
